# Association Between Plasma Sclerostin Levels and Body Mass Index in Women With Polycystic Ovary Syndrome

**DOI:** 10.7759/cureus.48875

**Published:** 2023-11-16

**Authors:** Mete Bertizlioglu, Ayse Gül Kebapcilar, Levent Kebapcilar, Fikret Akyürek, Zekiye Soykan Sert, Cetin Celik

**Affiliations:** 1 Department of Gynecology and Obstetrics, Konya City Hospital, Konya, TUR; 2 Department of Gynecology and Obstetrics, Medova Private Hospital, Konya, TUR; 3 Department of Endocrinology, Medova Private Hospital, Konya, TUR; 4 Department of Biochemistry, Selçuk University Medical School, Konya, TUR; 5 Department of Gynecology and Obstetrics, Aksaray University Medical School, Aksaray, TUR; 6 Department of Gynecology and Obstetrics, Selçuk University Medical School, Konya, TUR

**Keywords:** women, sclerostin, polycystic ovary syndrome, patients, body mass index

## Abstract

Background

Polycystic ovary syndrome (PCOS) is recognized as one of the most common endocrine pathologies in females of reproductive age worldwide. This study investigated the relationship between serum sclerostin levels and body mass index (BMI) in women with PCOS.

Methods

Women aged 18-40 years who presented to our clinic between January 1, 2019, and January 1, 2020, and were diagnosed with PCOS were included in this study. The patients' clinical and laboratory data were recorded, and waist circumference, hip circumference, and BMI values were calculated. The patients with a BMI of >25 kg/m^2^ were evaluated as Group I, those with a BMI of <18.5 kg/m^2^ as Group II, and those with a BMI of 18.5-25 kg/m^2^ as Group III. Serum sclerostin levels were compared between the BMI groups.

Results

The study included 90 patients. The mean BMI values were 32.2±2.1, 17.0±0.9, and 22.9±2.1 kg/m^2^ for Groups I, II, and III, respectively. We detected a statistically significant difference in serum sclerostin levels between Group II and Group I (p<0.005). There was a significant, positive correlation between the sclerostin level and BMI (r=0.258, p=0.014), weight (r=0.237, p=0.044), waist measurement (r=0.225, p=0.045), and hip measurement (r=0.225, p=0.033).

Conclusion

This study revealed that abnormal body composition in PCOS could alter circulating sclerostin levels.

## Introduction

Polycystic ovary syndrome (PCOS) is a condition characterized by hormonal imbalances in the female reproductive system [1]. Although the main pathophysiological mechanism of PCOS appears to be insulin resistance and androgen excess, its multifactorial etiology has not been fully elucidated [2]. PCOS cases carry a risk for additional diseases such as metabolic syndrome, dyslipidemia, type 2 diabetes, hypertension, and cardiovascular diseases [3]. The effect of PCOS on bone metabolism and skeletal mass remains controversial, with conflicting results having been reported from different studies in the literature [4-6]. While increased body mass index (BMI), hyperandrogenemia, hyperinsulinemia, and a relative increase in estrogen levels help protect women with PCOS against the development of osteoporosis and reduce the tendency for fractures, the presence of insulin resistance, low-grade chronic and estrogen deficiency, and inflammation may have a negative effect on bone metabolism in these patients [7].

Sclerostin is a protein that inhibits bone formation by acting on the Wnt pathway via LRP5/6 co-receptors [8]. The osteocyte-derived protein, sclerostin, prevents excessive bone formation by participating in the regulation of bone turnover. A previous study showed a positive correlation between serum sclerostin levels and age, BMI, and bone mineral content in healthy adults [9]. While PCOS is recognized for its hormonal and metabolic effects, its specific implications on bone health are not yet fully understood, and there is ongoing debate concerning how PCOS affects the Wnt pathway and sclerostin production. The current study aimed to evaluate the relationship between BMI and serum sclerostin levels in women with PCOS.

## Materials and methods

Study subjects and patient selection

This prospective study included women aged 18-40 years who presented to the gynecology and obstetrics clinic of Selçuk University Hospital of Konya between January 1, 2019, and January 1, 2020, and were diagnosed with PCOS. Approval for the study was received from the Clinical Research Ethical Committee of Selçuk University Faculty of Medicine (approval number: 2019/63). 

Patients under 18 and over 40 years of age, smokers, patients using medications that could affect ovarian function and lipid levels, those with calcium and vitamin D metabolism disorders, those with a history of chronic disease, those who had conditions manifesting with similar clinical features (e.g., Cushing's syndrome, androgen-secreting tumors, and congenital adrenal hyperplasia), and those who received hormonal drug therapy within the three-month period prior to the study were not included in the sample.

Study design

In all patients with PCOS, physical examinations were performed after taking anamnesis. All patients underwent pelvic ultrasonography for the evaluation of the uterus and bilateral ovaries. PCOS was diagnosed according to the Rotterdam criteria [10]. The waist circumference was measured at the midpoint between the lowest rib and the iliac crest. The hip circumference was measured from the widest part of the hip. BMI was calculated by dividing weight in kilograms by the square of height in meters. According to their BMI values, the patients were divided into three groups: BMI of >25 kg/m^2^ as Group I, BMI of <18.5 kg/m^2^ as Group II, and BMI of 18.5-25 kg/m^2^ as Group III. In all patients diagnosed with PCOS, luteinizing hormone (LH), follicle-stimulating hormone (FSH), estradiol (E2), dehydroepiandrosterone sulfate (DHEAS), total testosterone, sclerostin, 25-hydroxyvitamin D, fasting glucose, and fasting insulin levels were measured in venous blood samples collected after a 10-12-hour fasting period between the second and fifth days of menstruation. Additionally, the homeostatic model assessment for insulin resistance (HOMA-IR) index was calculated using the following formula: fasting insulin (μU/ml) x fasting glucose (mg/dl)/405 [11].

Biochemical analysis

All samples were obtained at our hospital in the morning after an overnight fast during the early follicular phase (second to fifth days) of the progesterone-induced or spontaneous menstrual cycle in PCOS. The DHEAS concentration was measured by chemiluminescent enzyme immunoassay. Testosterone measurements are performed by chemiluminescent immunoassay system using an IMMULITE 2000 immunoanalyzer (DPC-Siemens Healthcare Diagnostics, Deerfield, Illinois, United States). Fasting insulin levels were determined with a chemiluminescent method (Roche Diagnostics, Mannheim, Germany) utilizing an automatic immunoanalyzer. The serum blood samples were centrifuged, aliquoted, and directly frozen at -80°C for sclerostin analysis. Serum sclerostin levels were measured using an enzyme-linked immunosorbent assay kit (Cusabio, Wuhan, China).

Statistical analysis

IBM Statistical Package for Social Sciences (SPSS) Statistics for Windows, Version 22 (Released 2013; IBM Corp., Armonk, New York, United States) was used for statistical analysis. Data were presented as mean±standard deviation and median (minimum-maximum) values for continuous variables. Categorical variables were expressed as number (n) and percentage (%). Data were evaluated for normal distribution by the Kolmogorov-Smirnov test. Then, the analysis of variance or Kruskal-Wallis test was conducted to compare quantitative variables between groups. Baseline clinical and demographic characteristics were assessed using the Mann-Whitney U test or the independent sample t test. Correlations between variables were tested with non-parametric (Spearman's test for ordinal data) and parametric (Pearson's test for metric data) correlation analyses. We used stepwise models of multiple linear regression analyses to identify the independent predictors for serum sclerostin levels. The statistical significance level was accepted as p<0.05.

## Results

Ninety patients were included in the study and divided into three groups according to their BMI values: Group I (n=30), BMI of >25 kg/m^2^; Group II (n=30), BMI of <18.5 kg/m^2^; and Group III (n=30), BMI of 18.5-25 kg/m^2^. Table 1 presents the demographic and clinical characteristics of the cases. There was no significant difference between the three groups in terms of age, DHEAS, total testosterone, FSH, LH, E2, HOMA-IR, or fasting plasma glucose concentration (p>0.05). The mean BMI values were 32.2±2.1, 17.0±0.9, and 22.9±2.1 for Groups I, II, and III, respectively. Group I had a significantly higher sclerostin level than Group III (p=0.003). Although the sclerostin level of Group I was slightly higher than that of Group II, no statistically significant difference was found (p>0.05) (Table 2). Waist and hip measurements were significantly higher in Group I than in Groups II and III (Table 2). In the correlation analysis, the serum sclerostin level had a significant, positive correlation with BMI (r=0.258, p=0.014), weight (r=0.237, p=0.044), waist measurement (r=0.225, p=0.045), and hip (r=0.225, p=0.033) measurement (Table 3).

**Table 1:** Distribution of BMI groups according to clinical features in women with polycystic ovary syndrome Data are given as mean±standard error and median (minimum-maximum) or as number (percentage) FBG: fasting blood glucose; BMI: body mass index; HOMA-IR: homeostatic model assessment for insulin resistance; DHEAS: dehydroepiandrosterone sulfate; FSH: follicle-stimulating hormone; LH: luteinizing hormone; E2: estradiol

Variables	Group I (BMI of >25 kg/m^2^) (n=30)	Group II (BMI of <18.5 kg/m^2^) (n=30)	Group III (BMI of 18.5-25 kg/m^2^) (n=30)	p-value
Age	22.8±0.4	21.07±0.3	22.60±0.3	0.103
FBG (mg/dl)	86.5±9.0	84.0±7.2	85.6±11.1	0.541
Weight (kg)	83.9±8.0	48.2±5.3	60.5±6.5	<0.001
Waist circumference (cm)	97.7±7.5	69.1±5.8	79.1±8.6	<0.001
Hip circumference (cm)	117.9±6.2	90.7±4.5	97.5±8.2	<0.001
BMI (kg/m^2^)	32.2±2.1	17.0±0.9	22.9±2.1	<0.001
HOMA-IR	2.6 (1.2-4.3)	2.3 (1.5-3.5)	2.1 (1.2-3.8)	0.139
Insulin (µIU/mL)	16.2±11.7	6.9±2.4	10.1±3.9	<0.001
DHEAS (µg/mL)	275.8±127.6	285.0±121.7	254.2±137.4	0.637
Total testosterone (nmol/L)	0.6±0.4	0.5±0.2	0.5±0.2	0.514
Vitamin D3 (ng/ml)	12.3±5.5	13.9±6.3	11.9±5.6	0.382
FSH (mIU/L)	6.05±1.6	6.1±2.1	5.6±1.3	0.476
LH (mIU/L)	8.43±4.7	10.7±6.8	10.1±4.4	0.218
E2	37.7±13.98	35.5±13.74	37.7±15.60	0.411
Sclerostin (pmol/liter)	58.0±13.0	51.9±11.8	47.6±10.5	0.004

**Table 2:** Comparison of body composition characteristics between BMI groups in women with polycystic ovary syndrome Data are given as mean±standard error BMI: body mass index

Variables	Waist circumference (cm)	p-value	Hip circumference (cm)	p-value	Weight (kg)	p-value	Sclerostin (pmol/liter)	p-value
Group I vs. Group II	97.7±7.5 vs. 69.1±5.8	<0.001	117.9±6.2 vs. 90.7±4.5	<0.001	83.9±8.5 vs. 48.2±5.3	<0.001	58.0±13.0 vs. 51.9±11.8	0.114
Group I vs. Group III	97.7±7.5 vs. 79.1±8.6	<0.001	117.9±6.2 vs. 97.5±8.2	<0.001	83.9±8.5 vs. 60.5±6.5	<0.001	58.0±13.0 vs. 47.6±10.5	0.003
Group II vs. Group III	69.1±5.8 vs. 79.1±8.6	<0.001	90.7±4.5 vs. 97.5±8.2	0.001	48.2±5.3 vs. 60.5±6.5	<0.001	51.9±11.8 vs. 47.6±10.5	0.423

**Table 3:** Correlation between sclerostin levels and body composition characteristics in women with polycystic ovary syndrome BMI: body mass index

Variables	Sclerostin (pmol/liter)	
	r	p
Waist circumference (cm)	0.212	0.045
Hip circumference (cm)	0.225	0.033
BMI (kg/m^2^)	0.258	0.014
Weight (kg)	0.237	0.044

## Discussion

In the present study, we evaluated the association between BMI and serum sclerostin levels in patients with PCOS. We found a statistically significant difference in serum sclerostin levels between the group with a BMI of >25 kg/m^2^ and the group with a BMI of 18.5-25 kg/m^2^. The results of the study also revealed a positive correlation between sclerostin levels and waist and hip measurements in PCOS, suggesting that abnormal body composition in PCOS can alter circulating sclerostin levels.

The relationship between BMI and bone metabolism is complex and affected by many factors [12]. A meta-analysis reported a negative relationship between relative fat mass and bone mineral density in a population under 25 years of age [13]. This suggests that increased fat may cause structural deterioration in bone tissue. In addition, adipose tissue increases inflammation-related cytokines, such as tumor necrosis factor alpha and interleukin 6, which can suppress bone formation and stimulate bone resorption [14,15]. Sclerostin affects the modulation of bone turnover and bone formation by antagonizing the Wnt/β-catenin signaling pathway in osteoblasts and modulating RANKL levels that affect osteoclasts [8]. Bilen et al. [16] detected no statistically significant difference in sclerostin levels according to the presence of obesity or hirsutism in patients with PCOS. Another study demonstrated that serum sclerostin levels were associated with insulin resistance and nutritional status but not with sex hormone disorders in women with PCOS [17]. In the current study, we found that sclerostin levels in PCOS varied according to BMI. However, we did not observe a significant relationship between different body compositions and sex hormones.

In women with PCOS, insulin resistance is often caused by obesity, but it can also be present without obesity or overweight. Some studies have shown that insulin resistance may play a role in maintaining bone density in women with PCOS [18,19]. Hyperinsulinemia or insulin resistance can directly or indirectly stimulate the activity of osteoblast cells by suppressing the production of two binding proteins (sex hormone-binding globulin and insulin-like growth factor-binding protein). In the current study, the HOMA-IR index was high in patients with PCOS, but it did not statistically significantly differ between the BMI groups. Fasting insulin levels were significantly higher in obese women with PCOS. The present study found that there was no significant correlation between the sclerostin level and HOMA-IR and insulin levels.

This study has certain limitations. First, it was conducted at a single center with a limited number of patients. Second, since bone mineral densitometry could not be performed in women with PCOS included in the study due to ethical concerns and a lack of indications, the correlation between sclerostin and bone metabolism could not be evaluated. Finally, sclerostin was measured only in patients with PCOS, without forming a healthy control group.

## Conclusions

The results of this study revealed a statistically significant difference in serum sclerostin levels between women with PCOS who had a BMI value of >25 kg/m^2^ and those who had a BMI value of 18.5-25 kg/m^2^. There was a positive correlation between sclerostin levels and waist and hip measurements in PCOS. These results suggest that abnormal body composition may alter circulating sclerostin levels in these patients. Further studies are needed to demonstrate the effect of BMI on serum sclerostin levels in PCOS.

## References

[1] Escobar-Morreale HF (2018). Polycystic ovary syndrome: definition, aetiology, diagnosis and treatment. Nat Rev Endocrinol.

[2] Livadas S, Pappas C, Karachalios A (2014). Prevalence and impact of hyperandrogenemia in 1,218 women with polycystic ovary syndrome. Endocrine.

[3] Krishnan A, Muthusami S (2017). Hormonal alterations in PCOS and its influence on bone metabolism. J Endocrinol.

[4] Ganie MA, Chakraborty S, Sehgal A, Sreejith M, Kandasamy D, Jana M, Rashid A (2018). Bone mineral density is unaltered in women with polycystic ovary syndrome. Horm Metab Res.

[5] Katulski K, Slawek S, Czyzyk A (2014). Bone mineral density in women with polycystic ovary syndrome. J Endocrinol Invest.

[6] To WW, Wong MW (2012). A comparison of bone mineral density in normal weight and obese adolescents with polycystic ovary syndrome. J Pediatr Adolesc Gynecol.

[7] Karadağ C, Yoldemir T, Gogas Yavuz D (2017). Determinants of low bone mineral density in premenopausal polycystic ovary syndrome patients. Gynecol Endocrinol.

[8] Poole KE, van Bezooijen RL, Loveridge N, Hamersma H, Papapoulos SE, Löwik CW, Reeve J (2005). Sclerostin is a delayed secreted product of osteocytes that inhibits bone formation. FASEB J.

[9] Amrein K, Amrein S, Drexler C (2012). Sclerostin and its association with physical activity, age, gender, body composition, and bone mineral content in healthy adults. J Clin Endocrinol Metab.

[10] Azziz R (2006). Controversy in clinical endocrinology: diagnosis of polycystic ovarian syndrome: the Rotterdam criteria are premature. J Clin Endocrinol Metab.

[11] DeUgarte CM, Bartolucci AA, Azziz R (2005). Prevalence of insulin resistance in the polycystic ovary syndrome using the homeostasis model assessment. Fertil Steril.

[12] Savvidis C, Tournis S, Dede AD (2018). Obesity and bone metabolism. Hormones (Athens).

[13] Dolan E, Swinton PA, Sale C, Healy A, O'Reilly J (2017). Influence of adipose tissue mass on bone mass in an overweight or obese population: systematic review and meta-analysis. Nutr Rev.

[14] Braun T, Schett G (2012). Pathways for bone loss in inflammatory disease. Curr Osteoporos Rep.

[15] Reid IR, Baldock PA, Cornish J (2018). Effects of leptin on the skeleton. Endocr Rev.

[16] Bilen O, Bilen Y, Eroğlu M, Türkön H, Akdeniz Y, Asik M (2022). Serum sclerostin and Dickkopf-1 levels in patients with polycystic ovary syndrome [Article in Turkish]. Kocatepe Tıp Dergisi.

[17] Wyskida K, Franik G, Owczarek AJ (2020). Plasma sclerostin levels are associated with nutritional status and insulin resistance but not hormonal disturbances in women with polycystic ovary syndrome. Arch Gynecol Obstet.

[18] Yüksel O, Dökmetaş HS, Topcu S, Erselcan T, Sencan M (2001). Relationship between bone mineral density and insulin resistance in polycystic ovary syndrome. J Bone Miner Metab.

[19] Noyan V, Yucel A, Sagsoz N (2004). The association of bone mineral density with insulin resistance in patients with polycystic ovary syndrome. Eur J Obstet Gynecol Reprod Biol.

